# Association between Change in Normal Appearing White Matter Metabolites and Intrathecal Inflammation in Natalizumab-Treated Multiple Sclerosis

**DOI:** 10.1371/journal.pone.0044739

**Published:** 2012-09-17

**Authors:** Johan Mellergård, Anders Tisell, Olof Dahlqvist Leinhard, Ida Blystad, Anne-Marie Landtblom, Kaj Blennow, Bob Olsson, Charlotte Dahle, Jan Ernerudh, Peter Lundberg, Magnus Vrethem

**Affiliations:** 1 Neurology, Department of Clinical and Experimental Medicine, Division of Neuroscience, Faculty of Health Sciences, Linköping University, and Department of Neurology, County Council of Östergötland, Linköping, Sweden; 2 Division of Radiation Physics, Department of Medical and Health Sciences, Faculty of Health Sciences, Linköping University, and Department of Radiation Physics UHL, County Council of Östergötland, Linköping, Sweden; 3 Center for Medical Imaging and Visualization (CMIV), Linköping University, Linköping, Sweden; 4 Division of Radiology, Department of Medical and Health Sciences, Faculty of Health Sciences, Linköping University, and Department of Radiology UHL, County Council of Östergötland, Linköping, Sweden; 5 Clinical Neurochemistry Laboratory, Institution of Neuroscience and Physiology, Department of Psychiatry and Neurochemistry, The Sahlgrenska Academy, University of Gothenburg, Mölndal, Sweden; 6 Clinical Immunology, Department of Clinical and Experimental Medicine, Faculty of Health Sciences, Linköping University, Department of Clinical Immunology and Transfusion Medicine, County Council of Östergötland, Linköping, Sweden; 7 Neurology and Clinical Neurophysiology, Department of Clinical and Experimental Medicine, Faculty of Health Sciences, Linköping University, and Department of Neurology and Neurophysiology, County Council of Östergötland, Linköping, Sweden; Innsbruck Medical University, Austria

## Abstract

**Background:**

Multiple sclerosis (MS) is associated not only with focal inflammatory lesions but also diffuse pathology in the central nervous system (CNS). Since there is no firm association between the amount of focal inflammatory lesions and disease severity, diffuse pathology in normal appearing white matter (NAWM) may be crucial for disease progression. Immunomodulating treatments for MS reduce the number of focal lesions, but possible effects on diffuse white matter pathology are less studied. Furthermore, it is not known whether intrathecal levels of inflammatory or neurodegenerative markers are associated with development of pathology in NAWM.

**Methods:**

Quantitative proton magnetic resonance spectroscopy (^1^H-MRS) was used to investigate NAWM in 27 patients with relapsing MS before and after one year of treatment with natalizumab as well as NAWM in 20 healthy controls at baseline. Changes in ^1^H-MRS metabolite concentrations during treatment were also correlated with a panel of intrathecal markers of inflammation and neurodegeneration in 24 of these 27 patients.

**Results:**

The group levels of ^1^H-MRS metabolite concentrations were unchanged pre-to posttreatment, but a pattern of high magnitude correlation coefficients (r = 0.43–0.67, p<0.0005–0.03) were found between changes in individual metabolite concentrations (total creatine and total choline) and levels of pro-inflammatory markers (IL-1β and CXCL8).

**Conclusions:**

Despite a clinical improvement and a global decrease in levels of inflammatory markers in cerebrospinal fluid during treatment, high levels of pro-inflammatory CXCL8 and IL-1β were associated with an increase in ^1^H-MRS metabolites indicative of continued gliosis development and membrane turnover in NAWM.

## Introduction

Multiple sclerosis (MS) is a complex inflammatory disease of the central nervous system (CNS), causing demyelination and axonal damage already in the early stages of the disease course [1]. Treatments for MS aim to reduce CNS inflammation, considering it is the inflammatory response that at least in the initial phases of the disease drives the process leading to axonal damage. This view has been challenged by evidence that axonal damage, reflected in brain atrophy progression, may occur independently of focal inflammatory lesions [2]. Natalizumab (Biogen Idec Inc., Cambridge, MA, USA) prevents the migration of potential immune reactive cells into the CNS [3]. The result is reduced intrathecal inflammation [4], [5], [6], but whether natalizumab treatment also promotes axonal integrity is not known.

Magnetic resonance (MR) applications as proton magnetic resonance spectroscopy (^1^H-MRS) can provide additional information to conventional MR imaging (MRI) when studying subtle changes in brain tissue [7]. ^1^H-MRS may detect signs of axonal damage in what appears to be normal white (NAWM) and grey (NAGM) matter and allows for absolute quantification of specific metabolites (in mM aqueous concentrations) [8], [9], [10].

In this observational study of 27 patients with relapsing MS we used quantitative ^1^H-MRS to investigate changes in NAWM occurring during one year of treatment with natalizumab. The aims were (1) to detect possible changes in metabolite concentrations after treatment, determined using ^1^H-MRS, and (2) to assess whether changes in ^1^H-MRS detected metabolites were associated with ongoing intrathecal inflammation and neurodegeneration as measured by biomarkers in the cerebrospinal fluid (CSF).

## Methods

### Ethics Statement

The study was approved by The Regional Ethics Committee in Linköping (D nr M180-07 T130-09) and written consent was obtained from all participants.

### Patients

Natalizumab treatment (300 mg given intravenously once a month) was initiated in 27 patients with active MS (Table 1). Initiation of treatment was considered purely on clinical and MRI parameters, suggesting an active relapsing disease, and there was no prospectively followed placebo-treated group. Thus, the study design was observational. All included patients fulfilled the McDonald criteria of MS [11] and were consecutively recruited from the Department of Neurology at the University Hospital, Linköping. ^1^H-MRS examinations were performed before starting treatment with Natalizumab (mean 1.8 ± 2.0 months), *i.e.* ‘baseline’, and after one year of treatment (mean 12.5 ± 1.0 months), *i.e.* ‘follow-up’. Within the last four months before baseline ^1^H-MRS, 24 patients were on immunomodulatory treatment and 3 patients were not (details in Table 1). Sampling of CSF was obtained before start of natalizumab treatment (mean 1.8 ± 2.5 months) and after one year of treatment (mean 12.5 ± 1.4 months) in 25 patients since 2 patients refrained from lumbar puncture. The mean time from baseline ^1^H-MRS to sampling of CSF was 0.2 ± 2.0 months. Within the last four months before baseline sample of CSF, 22 patients were on immunomodulatory treatment and 3 patients were not. Among these 22 treated patients the washout period before baseline sample was one month for 2 patients, whereas 20 patients had no washout period at all. Ten patients had a relapse within 3 months before baseline ^1^H-MRS and CSF sampling, and two of these patients received high-dose methylprednisolone (1.0 g/day for 3 days). The effect of natalizumab on cytokine and chemokine levels in CSF for 12 patients participating in this study has been published elsewhere [5]. Neurological examination was done by a neurologist (CD or MV) and included definition of Expanded Disability Status Scale (EDSS) [12] and Multiple Sclerosis Severity Score (MSSS) [13] both at baseline and follow-up. The Symbol Digit Modalities Test (SDMT) [14] and the Multiple Sclerosis Impact Scale (MSIS-29) [15] were also performed at both occasions. For baseline comparisons, a control group of 20 healthy volunteers, 5 men and 15 women (median age 47.8 years, range 27–72), was examined with ^1^H-MRS at one occasion using the same MR scanner as the patient group.

**Table 1:** Patient and healthy control characteristics at baseline.

	Patients	HC
Number of subjects	27	20
Median age (years)	40.0 (range 22–62)	47.8 (range 27–72)***
Sex (M/F)	14/13	5/15
Median disease duration (years)^a^	9.3 (range 0.9–19.3)	NA
Diagnosis (RRMS / PRMS)	23/4	NA
EDSS (no of subjects)		NA
0–3.5	23	
4.0–5.5	2	
6.0–7.0	2	
Median EDSS	2.5 (range 0–7)	NA
Treatment b		NA
Interferon	20	
Glatiramer acetate	4	
Corticosteroids	3	
No treatment	3	
Median number of relapses last two years	2.0 (range 0–8)	NA
Number of patients with relapse last month before baseline CSF sample	3	NA

a Median number of years from first symptoms of MS to inclusion.

b Treatment within 4 months before baseline CSF sample.

c Three patients were treated with high-dose corticosteroids, in addition to interferon-β (2 patients) and glatiramer acetate (1 patient) respectively, due to relapses.

*** p<0.0005 compared with MS patients using independent samples t-test.

Abbreviations: RRMS = relapsing-remitting MS; PRMS = progressive MS with superimposed relapses; HC = healthy controls, EDSS = Expanded Disability Status Scale; CSF = cerebrospinal fluid, NA = not applicable.

### MR Data Acquisition

**Figure 1:**
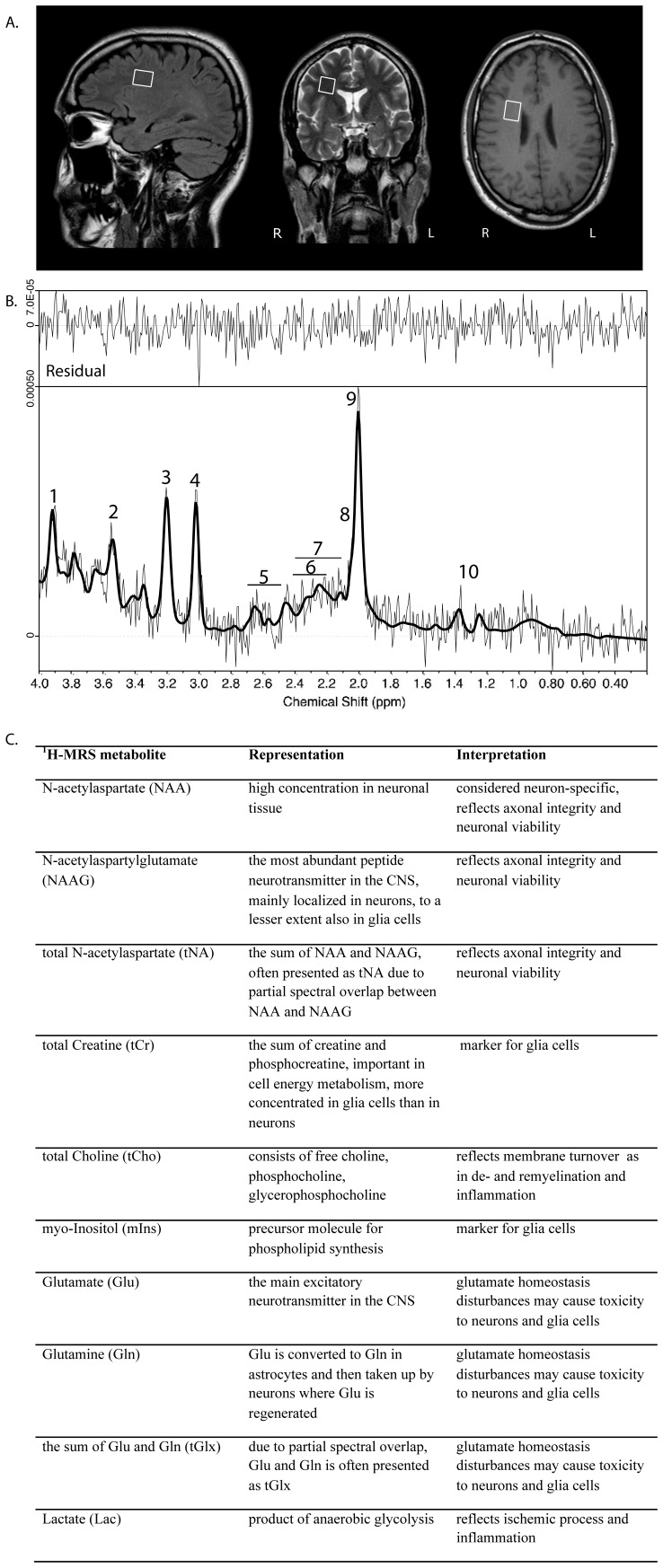
^1^H-Magnetic Resonance Spectroscopy (^1^H-MRS) examination with description of metabolites. A. Typical placement of a spectral voxel in normal appearing white matter. **B.** Typical ^1^H-MR spectrum with LCModel fit and residual of a natalizumab-treated MS patient (male, 30 years old). Spectral assignments: *1*, Cr-CH_2_-; *2*, mIns; *3*, Cho (CH_3_)_3_; *4*, Cr (CH_3_); *5*, NAA; *6*, Gln γ/β; *7*, Glu γ/β; *8*, NAAG-methyl; *9*, NAA-methyl; *10*, Lac CH_3_. **C.** Description of ^1^H-MRS metabolites, for reference see Dahlqvist O. [10].

All examinations were performed using an Achieva 1.5 T MR scanner (Philips, Best, The Netherlands), and an eight channels SENSE head coil. A T1 weighted axial, and a FLAIR sagittal, and a T2 weighted coronal image volumes were acquired for placing the MRS voxels. For quantification of R_2_ (1/T2) an axial ‘Multi Echo GRAdient echo and Spin Echo’ (ME-GRASE) pulse sequence was used to acquire data of the tissue water signal. The signals were acquired with a resolution of 3×2×2 mm^3^, 8 echoes with equidistant time-to-echo (TE) = 20–160 ms, TR = 5.28 s. In a later phase of the project (for the 7 last included patients) the ME-GRASE sequence was replaced with a ‘Multiecho acquisition of a saturation-recovery using Turbo spin-Echo Readout sequence’ (QRAPMASTER) [16] which enables simultaneous quantification of R_1_, R_2_ and PD. The signal was acquired using a resolution of 1×1×3 mm^3^, 4 echoes equidistant TE 14–66 ms, TR 2.7 s. MRS data were acquired using ‘Point RESolved Spectroscopy’ (PRESS), with an echo time of 30 ms and a repetition time of 3.0 s, 128 water suppressed spectra were averaged. In addition, eight spectra without water suppression were obtained as an internal reference standard. The MRS voxel was placed in periventricular NAWM (Figure 1A), lesions were avoided by freely rotating the voxel and adjusting the size (ranging 2–3 mL). A screenshot of the MRS voxel placement at baseline was saved and used for visual guidance at the follow-up examination. In the initial phase of the project (for the first 24 patients at baseline, and the first 9 patients at the one-year follow-up) a single MRS voxel was acquired. In a later phase of the project (for the last 3 patients at baseline, and the last 18 patients at the one-year follow-up), the examination time allowed for a second MRS voxel, which was added and placed using the same criteria, but lateral opposite to the first MRS voxel. The healthy controls (HC) were similarly examined using two MRS voxels (PRESS TR 30 ms, TR 3.0 s), which were placed bilaterally in parietal white matter. For quantification of R_2_ the QRAPMASTER sequence was used (TE = 14, 28, 42, 56 ms, TR = 3.0 s).

### Absolute Quantification of MRS Data

The MRS spectra were analyzed using LCModel ver 6.2-4G (S. Provencher, Canada) [17], with spectral basis set obtained from Dr M. Ljungberg at Sahlgrenska Academy (Gothenburg, Sweden). The internal water signal was used as an internal reference and absolute ‘aqueous fraction concentrations’ (mM aq.) were estimated using the method described previously [18]. The internal water R_2_ was estimated from the MEGRASE-volume or alternatively from the QRAPMASTER-volume, by least square fitting of the data to a mono-exponential function Y = M_0_ * exp(-R_2_ * t_TE_), where M_0_ is the T1 and a B_1_ weighted estimate of the proton density. The metabolites total creatine (tCr), total choline (tCho), myo-Inositol (mIns), total N-acetylaspartate (tNA), the sum of glutamate and glutamine (tGlx) and lactate (Lac) (Figure 1B–C) were analyzed [10].

### Exclusion Criteria of MRS Data

In order to avoid bias towards high concentrations, no measurements were excluded from the subsequent statistical analysis based on the SD % values in LCModel, according to the clear recommendations in the LCModel-documentation [19]. The spectra were visually inspected for improper residuals and large artifacts in the spectral region of interest (4.0–0.2 ppm). However, no spectra were excluded due to such residuals or artifacts.

### Biomarkers in CSF

Handling of CSF was done as previously described [5], and assessment of cytokine concentrations using an ultra-sensitive 10-plex multiple bead kit in accordance with the instructions provided (Invitrogen® Cytokine Ultrasensitive Human 10-plex Panel). An in-house assay was used to measure concentrations of CXCL10, CXCL11 and CCL22 [5]. The samples were analyzed on a Luminex^100^ instrument (Biosource), and the data were acquired using the StarStation 2.0 software (Applied Cytometry Systems, Sheffield, UK). Cytokine and chemokine values under the detection limit were assigned to half the value of the lowest standard point. CSF myelin basic protein (MBP) was analyzed using a sandwich ELISA (Active MBP ELISA, Diagnostic Systems Laboratories Inc., Webster, TX). The lower limit of detection for this assay is 0.1 ng/mL. CSF total tau (T-tau) and tau phosphorylated at threonine 181 (P-tau) were determined using the Luminex xMAP technology and the INNOBIA AlzBio3 kit (Innogenetics Zwijndrecht, Belgium) as previously described [20]. CSF neurofilament light protein (NFL) and glial fibrillary acidic protein (GFAP) were analyzed using previously described ELISA methods [21], [22].

## Statistics

### Baseline ^1^H-MRS Metabolites and Changes in Clinical Status, CSF Markers and ^1^H-MRS Metabolites in Patients Post- to Pretreatment

Differences in age and ^1^H-MRS metabolite concentrations between controls and MS patients at baseline were investigated using independent samples t-test. A possible association between age at baseline and baseline levels of ^1^H-MRS metabolites was investigated using non-parametric bivariate correlation analyses (Spearman). Logistic regression analyses were used to adjust for age differences between healthy controls and MS patients when comparing ^1^H-MRS metabolite concentrations. Wilcoxon signed rank test was used when comparing clinical status and CSF markers at baseline and follow-up. The changes in ^1^H-MRS metabolite concentrations were calculated for each MS patient as the paired difference between the one-year follow-up examination and the baseline examination. From the examinations where two MRS voxels were measured at baseline and/or follow-up, the mean concentration between these two voxels was used. Paired samples t -test was performed to investigate if any of the changes in ^1^H MRS metabolite concentrations were non-zero. One-way ANOVA test was used to investigate differences in baseline values of ^1^H-MRS in relation to treatment at baseline.

### Correlations during Treatment

Non-parametric bivariate correlation analyses (Spearman) were performed between changes in ^1^H-MRS detected metabolite concentrations during treatment versus CSF markers at baseline and follow-up. All data were explored in detail for a possible pattern of high magnitude correlation coefficients in a consistent direction.

All statistical calculations were performed in SPSS 20.0 software (SPSS inc., Chicago, IL). p < 0.05 was considered statistically significant.

### Plaque Contamination

The volume of plaque contamination in each MRS voxel was estimated by segmenting all plaques in the proximity of the MRS voxel. The segmentations were performed on T2w imaged by a neuroradiologist using MEVISLAB (MeVis Medical Solutions AG, Bremen, Germany). One MRS voxel contained more than 2 % of plaque. The observed significant correlations presented were still valid when statistical tests were redone excluding this potential outlier.

## Results

### Clinical Status and CSF Markers; Changes after One Year of Natalizumab Treatment

While on treatment with natalizumab, 24 patients were free from relapses, two patients had one relapse and one patient had two relapses. Two of these three patients had their relapse within two months of follow-up with ^1^H-MRS and one patient had a relapse within one month before follow-up CSF sampling. None of these patients received high-dose methylprednisolone. The annualized relapse rate decreased from 1.15 (calculated on data from the two years previous to baseline) to 0.15 on treatment. There was a significant decline in MSSS, MSIS-29 as well as in CSF total white blood cell counts and IgG index at follow-up compared to baseline (Table 2). Furthermore, MBP and NFL levels decreased significantly, whereas GFAP levels increased significantly (Table 2). The effect of natalizumab on NFL and GFAP levels for 9 patients participating in this study has been published elsewhere [23]. CSF levels of IL-1β (p = 0.003), IL-6 (p<0.05), and TNF (p = 0.09) decreased after one year of treatment, whereas CSF levels of GM-CSF, IFN-γ, IL-2, IL-4, IL-5, IL-10 were too low to be reliably detected by the method used (data not shown). CSF levels of CXCL8 (p = 0.02) CXCL10 (p<0.0005), CXCL11 (p<0.0005) and CCL22 (p<0.0005) decreased significantly after one year of treatment (data not shown). The effect of natalizumab on cytokine and chemokine levels in CSF for 12 patients participating in this study has been published elsewhere [5]. When excluding this subgroup of 12 patients from the analysis, there were still a significant decrease post-to pretreatment in levels of IL-1β (p = 0.04), CXCL10 (p = 0.002), CXCL11 (p = 0.003) and CCL22 (p = 0.002), whereas levels of IL-6, TNF and CXCL8 showed no significant change. No correlation was found between age of patient and baseline levels of CSF variables (total wbc count, IgG index, albumine ratio, neurodegenerative and inflammatory markers, data not shown). No differences in ^1^H-MRS detected metabolites at baseline were found between patients with different immunomodulating treatments at baseline (on group level, data not shown).

**Table 2:** Clinical and CSF data at baseline and at follow-up after one year of natalizumab treatment.

Clinical /CSF parameters	baseline	follow-up	p
EDSS	2.5 (0–7)	2.0 (0–6.5)	0.007
MSSS	3.9 (0.2–8.9)	3.1 (0.2–7.9)	<0.0005
MSIS-29			
Physical	2.2 (1.0–3.7)	1.5 (1.0–3.6)	<0.0005
psychological	2.1 (1.0–4.2)	1.6 (1.0–3.8)	<0.0005
SDMT	49 (5–69)	53 (3–74)	0.03
Total CSF wbc count^a^	2.2×10^6^ cells/L (0.2–31.0)	1.0×10^6^ cells/L (0.0–3.6)	0.002
N<5×10^6^ /L			
IgG index^a^	0.83 (0.48–4.06)	0.75 (0.45–3.84)	0.001
N<0.7			
Albumin ratio^a^	4.2 (2.2–11.4)	4.6 (2.2–10.1)	0.9
N<0.78			
Oligoclonal IgG bands^a^	23/25	24/25	NA
MBP b	1.08 ng/mL (0.67–4.20)	0.97 ng/mL (0.55–1.86)	<0.0005
NFL	509 ng/L (189–6006)^c^	289 ng/L (121–1872)^b^	<0.0005
GFAP	314 ng/L (164–710)^b^	382 ng/L (214–870)^b^	0.004
T-tau	43 ng/L (26–150)^d^	42 ng/L (25–155)^c^	0.1
P-tau	14 ng/L (5–33)^d^	14 ng/L (9–25)^c^	0.6

Median values are given and range within parenthesis. n = 27 unless stated otherwise. p refers to Wilcoxon signed rank test comparing baseline and follow-up.

a n = 25 since lumbar puncture was not done in two patients.

b n = 24,

c n = 23,

d n = 22 (n was reduced from 25 due to technical errors or lack of sample volume).

Abbreviations: CSF = cerebrospinal fluid; EDSS = Expanded Disability Status Scale; MSSS = Multiple Sclerosis Severity Score; MSIS-29 = Multiple Sclerosis Impact Scale; SDMT = Symbol Digit Modalities Test; wbc = white blood cell; MBP = myelin basic protein; NFL = neurofilament light protein; GFAP = glial fibrillary acidic protein; T-tau = total tauprotein; P-tau = phosphorylated tauprotein; N = normal reference values; NA = not applicable.

### ^1^H-MRS Detected Metabolites; Baseline Levels and Changes after One Year of Natalizumab Treatment and Correlation with Clinical and CSF Parameters

There was a significant group level difference in several metabolite levels between healthy controls and MS patients at baseline (Table 3). In contrast, no significant group level changes in ^1^H-MRS detected metabolite concentrations among MS patients between baseline and follow-up (*i.e*. at 0 versus 1 year) were observed (Table 3). As a control, this result remained also when excluding 10 patients with relapse within 3 months prior to baseline ^1^H-MRS. Since the absolute change in metabolite levels differed substantially between different patients at follow-up, we found it prudent to analyze if those differences could be associated with inter-individual diversity in CSF markers. For this reason we also performed correlation analyses between change in ^1^H-MRS detected metabolite concentrations during treatment in relation to CSF markers at baseline and follow-up. The aim with this procedure was to find possible patterns of associations as measured by clusters of high magnitude correlation coefficients. A pattern of high (r>0.5) and relative high (r = 0.4–0.5) magnitude correlation coefficients were found between one-year change in metabolite concentrations of tCr and tCho and levels of IL-1β and CXCL8 (r = 0.43–0.67) (Table 4 and 5). Apart from this pattern a single relative high magnitude correlation coefficient was found between change in tCr versus baseline levels of MBP (r = 0.42, p = 0.04) (Table 4). No correlation was found between age of patient at baseline and baseline levels of ^1^H-MRS metabolites (data not shown). In contrast, there was a correlation between age of HC and levels of ^1^H-MRS metabolite tCr (r = 0.54, p = 0.014) and mIns (r = 0.57, p = 0.008).

**Table 3:** ^1^H-MRS metabolite concentrations at baseline and at follow-up after one year of natalizumab treatment (presented as units of mM aq).

	HC			MS		MS				
	baseline (n = 20)		p^a^	baseline (n = 27)		follow-up (n = 27)		change during treatment		p^b^
	mean	SD		mean	SD	mean	SD	mean difference	CI	
tNA	13.35	0.82	0.001	12.20	1.40	11.95	1.25	−0.25	−0.72, 0.22	0.3
tCr	6.82	0.38	0.5	6.91	0.55	6.93	0.46	0.02	−0.25, 0.29	0.9
tCho	2.59	0.31	0.02	2.83	0.34	2.83	0.26	0.00	−0.11, 0.10	0.9
mIns	5.95	0.94	0.004	7.08	1.60	6.91	1.16	−0.17	−0.70, 0.36	0.5
tGlx	10.56	1.60	0.003	12.42	2.47	12.33	2.11	−0.09	−1.26, 1.09	0.9
Lac	0.29	0.27	0.2	0.45	0.52	0.55	0.62	0.10	−0.21, 0.41	0.5

To the left the mean and standard deviation (SD) of metabolite concentrations among healthy controls (HC).

p^a^ refers to significance level with independent samples t-test comparing HC with MS patients at baseline. Significant differences were still valid when adjusting for age using logistic regression analyses. To the right the mean and the standard deviation (SD) of metabolite concentrations among MS patients are presented at baseline and at follow-up. At the far right the mean difference in metabolite concentrations post-to pretreatment are presented with a 95% confidence interval (CI).

p^b^ refers to significance level with paired samples t-test comparing levels of metabolites in MS patients post-to pretreatment.

Abbreviations: tCr = total creatine; tNA = total N-acetylaspartate; mIns = myo-inositol; tCho = total choline; tGlx = glutamate + glutamine; Lac = lactate.

**Table 4:** Map of correlation analyses (bivariate non-parametric) between change in ^1^H-MRS metabolite concentrations during one-year versus levels of CSF markers at baseline and follow-up.

^1^H-MRS change	Inflammation								Th1				Th2		Neurodegeneration									
	CXCL8		IL-1β		IL-6		TNF		CXCL10		CXCL11		CCL22		NFL		GFAP		T-tau		P-tau		MBP	
tNA																								
tCr	*	**	*	**																			*	
tCho		**	*	**																				
mIns																								
tGlx																								
Lac																								

The magnitude of all correlation coefficients (r) are shown.

** r>0.5;

* r = 0.4–0.5;

no asterisk r<0.4. CSF markers are subdivided into markers of inflammation, chemokines representing T-helper lymphocytes 1 (Th1) and 2 (Th2) and neurodegeneration.

Abbreviations: tCr = total creatine; tNA = total N-acetylaspartate; mIns = myo-inositol; tCho = total choline; tGlx = glutamate + glutamine; Lac = lactate; NFL = neurofilament light protein; GFAP = glial fibrillary acidic protein; T-tau = total tauprotein; P-tau = phosphorylated tauprotein; MBP = myelin basic protein.

**Table 5:** Detail of correlation analyses map (Table 4) showing one-year change in metabolite concentrations versus baseline (left column for each analyte) and follow-up (right column for each analyte) levels of CXCL8 and IL-1β.

^1^H-MRS change		CXCL8 (n = 24)		IL-1β (n = 24)	
		Baseline	follow-up	baseline	follow-up
tNA	r	0.01	−0.04	−0.02	−0.15
	p	1.0	0.9	0.9	0.5
tCr	r	**0.48**	**0.67**	**0.49**	**0.52**
	p	0.02	<0.0005	0.02	0.01
tCho	r	0.38	**0.56**	**0.43**	**0.52**
	p	0.1	<0.01	0.03	0.01
mIns	r	0.21	0.09	0.30	0.10
	p	0.3	0.7	0.2	0.6
tGlx	r	−0.26	−0.14	−0.25	−0.25
	p	0.2	0.5	0.2	0.2
Lac	r	0.14	−0.004	0.09	−0.04
	p	0.5	1.0	0.7	0.8

High magnitude correlation coefficients in bold. r = correlation coefficient, p = significance level.

Abbreviations: tCr = total creatine; tNA = total N-acetylaspartate; mIns = myo-inositol; tCho = total choline; tGlx = glutamate + glutamine; Lac = lactate; NFL = neurofilament light protein; GFAP = glial fibrillary acidic protein; T-tau = total tauprotein; P-tau = phosphorylated tauprotein; MBP = myelin basic protein.

## Discussion

This is, to our knowledge, the first report in which quantitative ^1^H-MRS of NAWM is assessed in relation to intrathecal markers of inflammation and neurodegeneration. No change in ^1^H-MRS detected metabolite concentrations on a group level post- to pre-treatment was detected in natalizumab-treated MS patients. In contrast, high CSF levels of IL-1β and CXCL8 were associated with increasing levels of ^1^H-MRS metabolites indicative of glia cells (tCr) and membrane turnover (tCho).

This observational study had a longitudinal design that allowed us to correlate changes in ^1^H-MRS metabolite concentrations over one year with levels of biomarkers in CSF. The rationale for this procedure was that the change in metabolite concentrations often differed substantially between different patients at baseline and follow-up, resulting in no overall change in metabolite concentrations post-to pretreatment when analyzed on a group level. Therefore we wanted to determine if those changes between patients instead could be associated with individual diversity in the levels of CSF markers for inflammation and neurodegeneration. Accordingly, we sought all data for patterns of associations in a specific direction as measured by clusters of high magnitude correlation coefficients. This approach enabled an explorative search for associations and simultaneously managing the risk of overestimations of associations. We found a consistent pattern of high magnitude correlation coefficients indicating an association between change in specific ^1^H-MRS metabolite concentrations and the inflammatory markers IL-1β and CXCL8. In specific, intrathecal levels of IL-1β and CXCL8, the latter previously known as IL-8, correlated positively at baseline and at follow-up to change in levels of tCr (IL-1β and CXCL8) and tCho (IL-1β and for CXCL8 at follow-up only) over one year. These results suggest that patients, despite treatment, being characterized by high levels of inflammatory markers at follow-up, experienced an increase in density of glia cells and also increased membrane turnover in the MRS voxel during the one-year follow-up time. Inversely, elevated levels of inflammatory markers at baseline could possibly be a prognostic factor for the development of increased glia cell density and membrane turnover. Data should however be interpreted with caution, but since we observed a pattern of high magnitude correlation coefficients in a consistent direction we believe that these findings are not merely due to chance.

Increased tCho is a marker of augmented cell membrane turnover indicating enhanced de/remyelination, which also is in line with ongoing inflammation and increased levels of pro-inflammatory cytokines/chemokines [24]. The highest concentrations of tCr are found within astrocytes and oligodendrocytes [25] and tCr seems to represent gliosis rather than altered energy metabolism [26]. In a meta-analysis of ^1^H-MRS data on MS patients it was concluded that there was an overall increase in tCr levels in NAWM, whereas tCr levels in lesional white matter did not differ compared to controls [27]. In accordance, in one report combining immunopathologic findings with MRS, the concentrations of tCr in MS-lesions were not different from that of healthy controls, whereas tCr values were increased in the NAWM of a patient with glial abnormalities, as evidenced by concomitant augmentation of tCho and mIns [28]. Based on these reports, we interpret the elevated tCr as a glial cell proliferation and/or tissue migration in NAWM due to morphological changes (such as atrophy). Since mIns, as tCr, is considered a marker of glia cells, it is worth pointing out that no firm association between one-year change in mIns concentrations to levels of IL-1β and CXCL8 was observed, which one could argue should be demonstrated if both tCr and mIns represented gliosis. However, we propose that the explanation for this observation may be that concentrations of mIns are possibly more stable over time compared to tCr, and therefore no association with concentration change could be determined. This view is supported by a prospective study over two years on contrast-enhancing MS lesions in which absolute concentrations of tCr were shown to increase significantly over time whereas concentrations of mIns did not show any systematic changes [29].

Our observation of a pattern of high magnitude correlation coefficients between pro-inflammatory cytokine/chemokine levels and change in tCr during treatment may reflect that the process of gliosis in NAWM is associated with inflammation. In accordance, a transgenic mouse model with over-expression of the pro-inflammatory cytokine IL-6 in astrocytes, showed evidence of parenchymal inflammation as well as of gliosis, neuronal damage and axonal degeneration [30]. IL-1β, which here was associated with change in tCr, is a key pro-inflammatory cytokine. IL-1β is well known to recruit immune cells to the site of inflammation as well as to activate resident CNS immune cells like microglia [31]. Activation of microglia is further demonstrated to occur in NAWM adjacent to MS lesions and in NAWM of progressive states of MS [32], [33]. Since patients in the present study in general declined distinctly in clinical disease activity during natalizumab treatment, our findings speculatively indicate a subclinical inflammatory state that might be involved in the disease process affecting NAWM in spite of therapy. The hypothesis that diffuse NAWM gliosis development, possibly under the influence of inflammation, precedes axonal damage is also discussed in a ^1^H-MRS report on relapsing MS patients in early stages of the disease [34]. The authors’ interpretation of their data was that these patients showed signs of ongoing gliosis in NAWM in the absence of axonal damage even during clinical remission and despite immunomodulatory treatment. Another explanation to the association between levels of IL-1β and CXCL8 with change in ^1^H-MRS metabolites tCr and tCho may be dysfunctional astrocytes. There are reports indicating that astrocytes may secrete pro-inflammatory mediators as IL-1β and CXCL8 and thus could be the source of these mediators in CSF [35], [36], [37]. In that perspective, dysfunctional astrocytes instead of a primarily inflammatory response may contribute to intrathecal levels of pro-inflammatory mediators that we observed.

At baseline we found significant differences in NAWM between MS patients compared to HC; reduced levels of tNA and increased levels of mIns, tCho and Glx. These differences are in line with earlier reports, although there are some divergent results reported previously. As to NAA and tNA (the sum of NAA and NAAG), representing neuron viability, both decreased and unchanged levels compared to controls in cross-sectional studies have been reported [27], [38], [39]. Concentrations of tNA have also been shown to fluctuate over time, emphasizing the dynamic nature of metabolite status in NAWM and that changes could be reversible indicating neuronal dysfunction rather than loss [40]. In particular with respect to mIns but to some extent also concerning tCho, several different studies have demonstrated elevated metabolite concentrations in MS patients compared to controls [38], [39], [41], [42], [43] . mIns is considered to be a glia cell marker and tCho reflects membrane turnover and inflammation [44], [45]. Finally it has been reported that Glu, which together with Gln constitutes the Glx spectral resonances, is increased in acute lesions as well as in NAWM of MS patients compared to controls, indicating an excitotoxicity effect of this metabolite in the CNS [46]. Thus, taken together our findings of metabolite concentration differences at baseline between MS patients and controls are well in line with the literature and further support the assertion that the detected ^1^H-MRS metabolites in NAWM and measured differences are clinically relevant.

Only very limited and divergent results regarding the effects of disease-modifying treatments on ^1^H-MRS detected metabolites [47], [48] are available, and since the present, as well as most previous studies lack longitudinal healthy as well as placebo-based control groups, we cannot make conclusions on the specific effect of one-year natalizumab treatment on metabolite levels. However, regarding changes of metabolite concentrations in the normal brain there are data demonstrating that ^1^H-MRS metabolite concentrations do not change significantly over a 2-year period in the healthy human brain [49], and hence the differences between MS patients and controls in the present study would remain also after one year. Unfortunately (from a purely scientific point of view), it is for ethical reasons impossible to withhold a proven effective medication from MS patients with an active disease course, therefore making placebo studies unfeasible. However, in a prospective study of combined treatment with natalizumab and IFN-β1a compared to patients treated with IFN-β1a and placebo, no difference between NAA levels after one year was found [50]. In contrast, in the second year of treatment the authors reported a trend favouring treatment with natalizumab.

It is difficult, and sometimes impossible, to compare and interpret results from earlier reports on ^1^H-MRS detected metabolite levels since different investigators used quite different methodology for analyzing their data. For example ^1^H-MRS metabolite ratios with the most frequent denominator ‘Cr’ is often applied, assuming that Cr levels are stable in NAWM over time. Unfortunately this is not a recommended procedure as it has been shown that Cr is neither unaffected nor stable [29], [51]. Moreover, in a study by Vrenken et al the observed decline in NAA/Cr ratio in NAWM of all types of MS compared to controls arose entirely due to an increase in tCr concentration, whereas no difference in tNA was observed [38]. To avoid such obstacles when analyzing our data we therefore utilized absolute quantitative ^1^H-MRS to measure metabolite concentrations in this project.

Our group earlier reported a marked global decline in levels of cytokines and chemokines intrathecally after natalizumab treatment for one year [5]. As stated in the results, data on CSF levels of cytokines (IL-1β, IL-2, IL-4, IL-5, IL-6, IL-10, TNF, GM-CSF, IFN-γ) and chemokines (CXCL8, CXCL10, CXCL11, CCL22) from 12 of the patients in that study contributed to cytokine and chemokine data in the present report. In conformity with this earlier report we now found again that levels of IL-2, IL-4, IL-5, IL-10, GM-CSF and IFN-γ in CSF were too low to be detected, whereas levels of IL-1β, CXCL10, CXCL11 and CCL22 decreased significantly post- to pretreatment, even when analyzing levels only in patients not participating in the former study (n = 12). In contrast, levels of IL-6 and CXCL8 (former known as IL-8) in CSF showed no significant changes post-to pretreatment in this subgroup. We now also found that MBP levels in CSF decreased significantly at follow-up, which is interpreted as a decreased activity of demyelination intrathecally. NFL levels were also reduced at follow-up, which is in line with a recent Swedish multicenter study including 92 patients with relapsing MS, of whom nine patients from the present study participated [23]. The authors’ interpretation was that highly effective anti-inflammatory treatment reduces nerve injury in RRMS as measured by NFL levels. In the present study, we noted an increase in GFAP levels at follow-up, a finding that was not noted in the larger cohort, the discrepancy probably being due to differences in the patient material.

To summarize, no significant changes in ^1^H-MRS detected metabolite concentrations after one year of natalizumab treatment with a group level perspective were observed, despite an improvement in clinical scoring systems and a reduction in CSF markers for inflammation and neurodegeneration. In contrast, correlation analyses showed a pattern where levels of pro-inflammatory markers IL-1β and CXCL8 in the CSF were associated with changes in ^1^H-MRS metabolite concentrations indicative of gliosis and membrane turnover in NAWM, the meaning of which is uncertain but may speculatively indicate involvement of subclinical persistent inflammation in MS diffuse white matter pathology.
